# Inside *Mycobacterium bovis* SB0120 spoligotype circulating in Italy: analysis of the most frequent genotypes by whole genome sequencing

**DOI:** 10.3389/fmicb.2024.1416605

**Published:** 2024-07-26

**Authors:** Erika Scaltriti, Karaman Iyad, Maria Beatrice Boniotti, Ilaria Menozzi, Luca Bolzoni, Dorotea Ippolito, Flavia Pruiti Ciarello, Daniela Loda, Mario D’Incau, Mariagrazia Zanoni, Vincenzo Di Marco Lo Presti, Piera Mazzone, Stefano Gavaudan, Maria Lodovica Pacciarini

**Affiliations:** ^1^Risk Analysis and Genomic Epidemiology Unit, Istituto Zooprofilattico Sperimentale Della Lombardia e Dell'Emilia-Romagna (IZSLER), Parma, Italy; ^2^National Reference Centre for Bovine Tuberculosis, Istituto Zooprofilattico Sperimentale della Lombardia e dell'Emilia Romagna – IZSLER, Brescia, Italy; ^3^Area Territoriale Barcellona Pozzo di Gotto, Istituto Zooprofilattico Sperimentale della Sicilia "A. Mirri", Barcellona Pozzo di Gotto, Messina, Italy; ^4^Istituto Zooprofilattico Sperimentale dell’Umbria e delle Marche “Togo Rosati” (IZSUM), Perugia, Italy; ^5^Istituto Zooprofilattico Sperimentale dell’Umbria e delle Marche “Togo Rosati” (IZSUM), Ancona, Italy

**Keywords:** whole genome sequencing, bovine tuberculosis, *Mycobacterium bovis*, phylogeny, spoligotypes, VNTR-MIRU genotypes, SNPs

## Abstract

Bovine tuberculosis (bTB) is a chronic inflammatory disease primarily caused by *Mycobacterium bovis*. The infection affects domestic animals and wildlife, posing a zoonotic risk to humans. To understand the dynamics of transmission and genetic diversity in Italy’s *M. bovis* population, we conducted whole-genome sequencing (WGS) analysis on two prevalent genotypes, belonging to Spoligotype SB0120, identified in different geographical and temporal contexts. By comparing these genomes with international *M. bovis* isolates, we identified a distinct clade within the lineage La1.2, encompassing the Italian SB0120 isolates, indicating a genomic segregation of Italian *M. bovis* from other European isolates. Within Italy, a significant level of genetic variability emerged across regions, while isolates within epidemiologically linked outbreaks exhibited minimal genetic diversity. Additionally, isolates derived from cattle and wild boars within a tuberculosis hotspot in Central Italy and from cattle and black pigs in Sicily formed unified clonal clusters. This indicates the presence of persistent strains circulating in the examined regions. The genetic diversity within herds was limited, as specific clones endured over time within certain herds. This research enhances our comprehension of the epidemiology and transmission patterns of bTB in Italy, thereby aiding the development of precise control strategies and disease management. Using WGS and implementing standardized protocols and databases will be pivotal in combating bTB and promoting One-Health approaches to address this noteworthy public health concern.

## 1 Introduction

Bovine tuberculosis (bTB) is a chronic inflammatory disease mainly caused by *Mycobacterium bovis* and, to a lesser extent, by *Mycobacterium caprae*. The infection can affect several domestic animals and wildlife species and has zoonotic relevance owing to its potential transmission to humans.

*Mycobacterium bovis* is a component of the *Mycobacterium tuberculosis* complex (MTBC) (Huard et al., 2006; Smith et al., 2006), characterized by 99,9% similarity at the nucleotide level and identical 16S rRNA sequences (Huard et al., 2006). MTBC is composed of highly clonal species with no evidence of genetic material transfer between lineages (Smith, 2012). MTBC derived from a common ancestor related to *Mycobacterium canettii* (Blouin et al., 2012), which includes different members: *M. tuberculosis, M. bovis, M. africanum, M. microti, M. caprae, M. canettii, M. pinnipedii, and M. origys* (Brosch et al., 2002; Rodriguez-Campos et al., 2012; van Ingen et al., 2012). It was suggested that members of the MTBC may be considered a series of host-adapted variants designated “ecotypes” of *M. tuberculosis* rather than species (Smith et al., 2006, 2009).

Four clonal complexes of *M. bovis* were defined based on distinct spoligotypes (Kamerbeek et al., 1997), Single Nucleotide Polymorphisms (SNPs) signatures, and/or specific deletions ranging from 806 to 14,094 bp: European 1 (Eu1), European 2 (Eu2), African 1 (Af1), and African 2 (Af2) (Müller et al., 2009; Berg et al., 2011; Rodriguez-Campos et al., 2012; Smith, 2012). However, these clonal complexes do not represent the whole diversity of *M. bovis* genomes (Zimpel et al., 2020). Recently, Branger et al. (2020) proposed the existence of the clonal complex “European 3,” corresponding to the “cluster I” described by Hauer et al. (2019), mainly constituted by spoligotype SB0120, which is one of the most common spoligotypes of *M. bovis* responsible for bTB outbreaks in Italy, France, Germany, Zambia, and Algeria (Haddad et al., 2004; Boniotti et al., 2009; Munyeme et al., 2009; Sahraoui et al., 2009; Smith, 2012; Ghavidel et al., 2018). SB0120 is considered the ancestral spoligotype of *M. bovis* since it is generally accepted that spoligotype patterns evolve by loss (deletion) of spacer sequences of the DR locus (Rodríguez et al., 2010; Smith, 2012).

Genetic differentiation of strains has become an indispensable tool to study the evolution, epidemiology, and ecology of this pathogen, providing a means to detect and characterize the spread of pathogens in domestic livestock and establish eventual spread in the wildlife population.

Moreover, molecular typing has been used in many countries as a supporting tool to understand epidemiological links among outbreaks and to conduct efficient bTB control strategies. The most popular approach for a long time has been a combination of spoligotyping and Variable Numbers of Tandem Repeats-Mycobacterial Interspersed Repetitive Unit (VNTR-MIRU) with different markers selected for their high discrimination capacity and based on the MTBC population present in the territory (Boniotti et al., 2009; Gormley et al., 2014). Couvin et al. (2022) performed a global evaluation of the discriminative capacity of 12- and 15-loci VNTR-MIRU panels using data from the SITVITBovis database.^1^

In Italy, a study, performed on 1,560 *M. bovis/M. caprae* strains, allowed to select a panel of 12 VNTR-MIRU loci, which showed the most discriminative ability for VNTR-MIRU typing (Boniotti et al., 2009). Based on this study, molecular typing (spoligotyping/ VNTR-MIRU) has been applied since 2008 on *M. bovis/M. caprae* isolates collected in Italy, providing a national animal database (ITAN-TB) containing information about genotypes, species, and herd of origin, representing an important tool for epidemiological purposes.

Traditional genotyping provided preliminary information on the genetic characteristics of the *M. bovis* population in Italy and other countries, including the presence of *M. caprae* in more than 8% of bTB outbreaks, the low frequency of British Isle spoligotypes corresponding to EU 1 (Boniotti et al., 2009; Smith, 2012), and of SB0121, corresponding to the clonal complex EU 2, common in France, Portugal, and Spain (Duarte et al., 2010; Rodríguez et al., 2010; Hauer et al., 2016), the dominant presence of SB0120, described as the most prevalent (20–25%) in continental European countries (France, Germany, Belgium) (Kubica et al., 2003; Haddad et al., 2004; Hauer et al., 2016), and reported in approximately 50% of bTB outbreaks in Italy (Boniotti et al., 2009).

Although traditional typing performed with spoligotyping/VNTR-MIRU is a worldwide recognized tool for epidemiological investigation (Hauer et al., 2019), recent studies show the limitations of these methods in tracing the origin of a given infection (Rodriguez-Campos et al., 2011; Guimaraes and Zimpel, 2020). In addition, even if these typing techniques target polymorphic genetic regions, they interrogate less than 1% of the genome and therefore have an intrinsically restricted discriminatory power (Hauer et al., 2019). Furthermore, homoplasy phenomena can occur in DR region (Corner et al., 1995; Smith, 2012). and VNTR loci (Comas et al., 2009) giving rise to identical patterns in separate sub-lineages (Comas et al., 2009; Trewby et al., 2016).

Although the estimated substitution rates of *M. bovis* range from 0.15 to 0.53 single nucleotide variants per genome per year (Trewby et al., 2016; Guimaraes and Zimpel, 2020), the comparison of entire genomes using Whole Genome Sequencing (WGS) has shown a high capacity of differentiation of *M. tuberculosis* and *M. bovis* strains (Biek et al., 2012; Homolka et al., 2012; Joshi et al., 2012; Roetzer et al., 2013; Luo et al., 2014). Accordingly, WGS has been demonstrated to be useful for the differentiation of *M. tuberculosis* strains with identical VNTR-MIRU profiles (Guimaraes and Zimpel, 2020).

In Italy, the combined use of spoligotyping and VNTR-MIRU (Boniotti et al., 2009) has highlighted the presence of high frequent profiles isolated in several herds, in different spatiotemporal contexts, apparently without any epidemiological correlation based on the available information.

Furthermore, WGS can improve the accuracy of the phylogenetic and epidemiological analysis of geographically characteristic genotypes over time (Lasserre et al., 2018, Orloski et al., 2018, Price-Carter et al., 2018, Hauer et al., 2019) and provide additional insights into the presence of new clonal complexes or lineages (Hauer et al., 2019; Kohl et al., 2020; Zimpel et al., 2020).

In the last few years, several genomic studies on *M. bovis* national relevant clones and populations have been published, allowing for the description of the phylogenetic and phylodynamic relationships among *M. bovis* isolates circulating in Europe and around the world (Hauer et al., 2019, Loiseau et al., 2020, Zwyer et al., 2021, Pozo et al., 2022). Regarding the *M. bovis* Italian population, very little is known about the association of specific clonal groups (Hauer et al., 2019) or global lineages (Loiseau et al., 2020; Zimpel et al., 2020) with the VNTR-MIRU genotypes circulating in Italy.

The present study aimed to employ SNP analysis based on WGS to identify accurate phylogenetic relationships among isolates belonging to the most frequent *M. bovis* SB0120/VNTR-MIRU profile in Italy, which originates from various epidemiological contexts and distinct geographical regions. Moreover, we compared their sequences with the SB0120 genomes already available and with the main sub-lineages circulating in Europe.

The most frequent SB0120/VNTR-MIRU profile circulating in Italy was selected based on a previous large-scale spoligotyping/VNTR-MIRU genotyping study (Boniotti et al., 2009) (Pacciarini, unpublished results). Our study includes: (1) isolates from outbreaks that were very distant geographically and temporally and (2) isolates that have been circulating for many years in the same restricted geographical area both in cattle and wild species.

Furthermore, this analysis aims to assess the genetic diversity within and between regions. Moreover, it seeks to examine genetic diversity within epidemiologically correlated outbreaks and within individual herds.

This genomic study will contribute to a more accurate description of the phylogenetic relationships between Italian and European *M. bovis* isolates of the SB0120 spoligotype, providing additional insight into the transmission and surveillance of bTB in Italy, defining molecular lineages (Hauer et al., 2019, Zimpel et al., 2020, Zwyer et al., 2021) as well as new clonal targeting profiles circulating in Italy.

## 2 Materials and methods

### 2.1 Microbiological and molecular characterization of *Mycobacterium bovis* isolates collected in ITAN-TB

As a national reference Centre for Bovine Tuberculosis (CRN-TB), we collected more than 7,000 *MTBC* isolates from 2000 to 2020 in Italy. The collection, called ITAN-TB, contains *M. bovis/M. caprae* strains mainly isolated from cattle and buffalo bTB outbreaks during the eradication program. The ITAN-TB database is not public, but its data can be requested by Italian authorities or the network of national laboratories for conducting epidemiological investigations.

All the isolates were identified by microbiological methods (Metchock et al., 1999) and by molecular methods described by Kulski et al. (1995). Mycobacteria of the MTBC were identified by PCR/RFLP of the *gyrB* gene, as reported by Boniotti et al. (2014), and by two High Resolution Melting (HRM) assays targeting the *gyrB* gene. The two assays were developed to identify *M. tuberculosis*, *M. microti, M. bovis,* and *M. caprae*: the first reaction allows distinguishing *M. tuberculosis*, *M. bovis/M. caprae*, and *M. microti*, while the second differentiates *M. bovis* from *M. caprae*. The first pair of primers, consisting of myc_HRM_1F (5’-GTCGAGATCAAGCGCGAC-3′) and myc_HRM_1R (5’-TTCGAAAACAGCGGGGTCG-3′), amplifies a fragment of 135 base pairs (bp), while the second pair of primers composed of TBCX_2F (5’-TGACGATATCCGGGAAGGC-3′) and TBCX_2R (5’-TCAAACCAGTGGGTCAGCTG-3′), amplifies a fragment of 150 bp. The reaction was performed in a total volume of 20 μL. One μl of DNA from the sample or control was added to a reaction mixture containing 7 μL of sterile H_2_O, 10 μL of 2X mastermix (Bio-Rad), 1 μL of Primer Forward 10 μM, 1 μL of Primer Reverse 10 μM. Reference strains of *M. tuberculosis, M. microti*, *M. bovis,* and *M. caprae* were included as melting curve standards and positive controls in each experiment. The amplification was carried out with an initial activation of the DNA polymerase for 3 min at 98°C, followed by 50 cycles divided into the following two steps: 98°C for 5 s and 60°C for 10 s. A post-amplification cycling step was performed with a denaturation step at 95°C for 1 min and renaturation at 65°C for 1 min followed by an HRM ramping from 65°C to 95°C with an increment of 0,2°C every 10 s. The HRM assay was performed on the CFX96^™^ Real-Time PCR Detection System (Bio-Rad) with the SsoFast^™^ EvaGreen^®^ Supermix (Bio-Rad). The software used for the analysis of amplification and melting curves are CFX Maestro (Bio-Rad) v2.2 and Precision Melt Analysis^™^ (Bio-Rad) v1.3., respectively.

From 2008, *M. bovis/M. caprae* strains were typed systematically by spoligotyping (Kamerbeek et al., 1997) and by VNTR-MIRU with ETRA-E (Frothingham and Meeker-O’Connell, 1998) and 7 additional VNTR/MIRU markers selected in the study of Boniotti et al. (2009), obtaining more accurate genetic profiles. Spoligotyping was performed as described by Kamerbeek et al. (1997). The spacer sequences contained in the direct repeat locus were detected by hybridization onto a spoligotyping membrane (Mapmygenome, Hyderabad, India). Spoligotypes were named according to an agreed international convention by using the www.mbovis.org database (Smith, 2012). VNTR-MIRU typing was performed on 12 VNTR-MIRU loci: ETRA (2165), ETRB (2461), ETRC (577), ETRD (580), ETRE (3192), Qub11a (2163a), Qub11b (2163b), Qub26 (4052), Qub1895 (1895), Qub15 (3155), Qub3232 (3232), and MIRU26 (2996) (Frothingham and Meeker-O’Connell, 1998; Supply et al., 2000; Roring et al., 2002; Skuce et al., 2002). The panel contains the 8 loci recommended by the European consortium SSPE-CT-2004-501903 Veterinary Network of Laboratories Researching into Improved Diagnosis and Epidemiology of Mycobacterial Diseases (VENoMYC).

### 2.2 Selection of *Mycobacterium bovis* strains for this study

To select the strains for this study, we evaluated the ITAN-TB database collection from 2008 to 2018, which contains mainly *M. bovis/M. caprae* strains isolated from bTB outbreaks during the eradication program and genotypes isolated from other domestic and wild species. Since nearly all the North and Centre of Italy achieved a disease-free status (DFS) in the last 10 years, the ITAN-TB mainly contains MTBC isolates from Southern regions, especially Sicily, Campania, Apulia, and Calabria, where bovine tuberculosis is still present. Therefore, most of the *M. bovis* strains analyzed in this study were isolated from these regions.

In this study, we selected a set of 76 SB0120 isolates, focusing on the most frequent genotype, based on VNTR-MIRU profile (Supplementary Tables S1, S3). In particular, we considered: (i) thirty-six strains belonging to the main VNTR-MIRU genotype circulating in Italy from different regions across Italy, including Campania, Sicily, Apulia, Calabria, Veneto, and Lazio. These strains, characterized by the genetic profile VNTR-MIRU 4,5,5,3,3,10,4,4,4,3,6,5, here named Italian Genotype 1, were representative of the Italian population (in red Supplementary Figure S1). They were chosen to reflect the temporal and regional distribution observed between 2008 and 2018, (ii) twenty-three strains, also belonging to the Italian Genotype 1, were specifically sampled from cattle and black pigs within a delimited geographic area of Sicily, around the Caronia municipality, which is located in the Nebrodi Park (with an area of nearly 860 km^2^ in the Messina province). Over a three-year period (2015–2018), these isolates were obtained from animals with well-documented epidemiological backgrounds (Supplementary Figure S2); Caronia, marked by its extensive farming systems and shared pastures, provided a unique setting for studying disease dynamics, and (iii) seventeen isolates, designated as the Matelica Clade and belonging to SB0120, were identified in cattle and wild boars originating from a geographically area (about 1,000 km^2^) around the Matelica municipality in the Macerata province over an eight-year span. These specimens exhibited the genetic profile VNTR-MIRU 3,3,5,3,3,10,4,4,4,3,6,5. They were collected from herds and hunting areas proximal to livestock farms in the Marche region of Central Italy. The epidemiological data associated with these cases of clustered outbreaks included comprehensive information such as outbreak identification records, the timing of the last IDT (intradermal tuberculin) test execution, details regarding shared pastures, animal movements during the interval between the last IDT test and the detection of positive cases, and genotype data. We included the genomes belonging to the Matelica Clade in the analyses with the aim to compare the genomic variability observed between samples originated from a unique introduction in a geographically restricted area (i.e., the Matelica Clade) with respect to the genomic variability observed within clades belonging to the Italian Genotype 1, which were associated, based on the epidemiological and genotypical information, to *M. bovis* introduction in areas with similar territorial extension (such as Caronia).

Moreover, from a total of 8 herds, 2 to 7 strains from each of these herds were selected and analyzed to assess genetic diversity within herds.

In addition, in the phylogenetic analysis, five *M. bovis* genomes characterized by genotypes different from the Italian Genotype 1 and Matelica Clade were included as outgroups: two strains isolated in Sicily characterized by spoligotype SB0841; one strain isolated in Campania with 2 repeat units in ETRD instead of 3; one strain isolated in Apulia with 3 different markers (Qub11a: 9, Qub1895: 2, Qub15: 2); one strain isolated in Apulia with 2 different markers: Qub11a: 9, Qub3232: 4 (See Supplementary Table S1).

### 2.3 DNA extraction

*Mycobacterium bovis* strains frozen in tryptic soy broth with 10% glycerol were thawed and cultured on 5 Stonebrink medium (prepared as solid slants in screw-cap tubes) for 3–4 weeks at 37°C. For each isolate, all the colonies were transferred into 500 μL of Middlebrook 7H9 broth and inactivated for 20 min at 95°C.

DNA was extracted by the Cetyltrimethylammonium bromide (CTAB) protocol as described by Ausubel et al. (2003) with minor modifications. In short, lipase was added to a final concentration of 11,3 μg/μl, and the tube was incubated overnight at 37°C. Fifty μl of proteinase K (20 mg/mL) and 1,5 mL of CTAB extraction solution were added, and the mixture was incubated for 1 h at 65°C. After centrifugation for 10 min at 12.500 g, the pellet was discarded, and an equal volume of chloroform was added to the recovered aqueous phase. The mixture was centrifuged as described above. The aqueous phase was recovered, mixed with 2X volume of CTAB precipitation solution [CTAB 0,5% (w/v), NaCl 40 mM], and incubated for 1 h at room temperature. After centrifugation, the aqueous phase was discarded, and the pellet was resuspended in 350 μL of NaCl 1,2 M. The mixture was extracted with chloroform as described above. The aqueous phase was recovered, and the solution was precipitated by adding 0,6 volume of isopropanol at room temperature. After washing the pellet with 500 μL of Ethanol 70% vol/vol, the pellet was resuspended in 30 μL of TE (Tris–HCl 10 mM, EDTA 1 mM pH8).

### 2.4 Whole genome sequencing and SNP analysis

Genomic libraries were prepared starting from extracted genomic DNA using the Nextera XT DNA Library Preparation Kit (Illumina), quantified by a Quantus Fluorometer (Promega, Fitchburg, United States), and sequenced on either Miseq or Nextseq 550 systems (Illumina) generating 2 × 250 and 2 × 150 bp paired-end reads, respectively. Raw reads were checked for quality using FastQC (Babraham Bioinformatics, n.d.) and for possible contaminants through Kraken2 software (Wood and Salzberg, 2014). Reads were trimmed with Trimmomatic ver. 0.38 (minimum mapping quality for an alignment to be used of 30 and minimum base quality for a base to be considered of 20) (Bolger et al., 2014) and used as input for SNP analysis.

To contextualize Italian isolates within the international framework, we performed a SNP analysis comprising *M. bovis* isolates sequenced in this study, along with a selection of representative La genomes from international databases. This selection includes a broad representation of La 1.2, to which the Italian isolates from the study of Zwyer et al. (2021) appear to belong (Supplementary Table S2). The SNP analysis was performed starting from trimmed reads with the CFSAN SNP pipeline ver. 0.8 (Davis et al., 2015) using the *M. bovis* AF2122/97_BX24833 genome (Accession N. AF2122/97_BX248333) *M. bovis* lineages/sub-lineages of Italian isolates were checked running KvarQ (Steiner et al., 2014) with the new KvarQ testsuite (MTBC_animals) indicated in Zwyer et al. (2021). A second analysis based on a sub-dataset (only *M. bovis* Italian isolates sequenced in this study plus outgroups) was also performed using the CFSAN SNP pipeline ver. 0.8 (Davis et al., 2015) with default settings except for the following parameters: minimum mapping quality for an alignment = 30 minimum base quality for a base = 20. More deeply, trimmed reads were mapped to the *M. bovis* Mb3601 genome, representative of a new clonal complex EU3 isolated in France and characterized by SB0120 spoligotype (Assembly Accession Number: LR699570.1) (Branger et al., 2020) using Bowtie2 (Langmead and Salzberg, 2012), and SNPs were identified using VarScan (Koboldt et al., 2012). Abnormal SNPs (i.e., SNPs from the 500 nt-edge regions of contigs and recombinant and repetitive regions with more than 3 SNPs per 1,000 bases) were removed. The high-quality core SNP matrixes obtained from both SNP analyses were used to infer phylogeny through a Maximum Likelihood (ML) algorithm using RaxML software with 100 bootstrap replicates (Stamatakis, 2014). Phylogenetic trees were edited using iTOL ver.6.^2^

## 3 Results

### 3.1 Spoligotypes and VNTR-MIRU genotypes in Italy

Since 2008, all the isolates collected in Italy have been genotyped by spoligotyping and VNTR-MIRU. Spoligotyping differentiated the 4,845 MTBC strains from 3,353 herd outbreaks collected between 2008 and 2020 into 136 spoligotypes (Table 1). Fourteen spoligotypes belonged to *M. caprae,* while 122 to *M. bovis*. The most frequent spoligotypes were SB0120 (44,4%), SB0134 (12,1%), SB0841 (10,7%) for *M. bovis,* and SB0418 (6,8%) for *M. caprae*. These spoligotypes represent 77% of bTB strains circulating in Italy. Seventy-one spoligotypes were found in only one outbreak.

**Table 1:** *Mycobacterium bovis/Mycobacterium caprae* population in Italy in 2008–2020 (*N* = 3,353 outbreaks).

Spoligotypes	%	VNTR-MIRU	%	Spoligo_VNTR-MIRU	%
SB0120	44,4	4553310444365	7	SB0120_4553310444365*	5,6
SB0134	12,1	5453410454365	3,9	SB0134_5453410454365	3,5
SB0841	10,7	5453310444365	3,9	SB0841_5453310444365	3
SB0418	6,8	3353310444365	3,2	SB0120_3353310444365**	2,6
SB0872	2,2	5353310444365	2,7	SB0120_5553410454365	1,5
SB0121	2,2	5553310444365	2,3	SB0841_555335444365	1,5
Other Spoligos	23	Other VNTR-MIRUs	77	Other Spoligo_VNTR-MIRUs	82,3

VNTR-MIRU order of markers: ETRA, ETRB, ETRC, ETRD, ETRE, Qub11a, Qub11b, Qub26, Qub1895, Qub15, Qub3232, MIRU26; *Italian genotype; **Matelica Clade.

The combination of spoligotyping/VNTR-MIRU identified a total of 1,035 genotypes, including 318 clusters of 2–190 isolates and 717 unique profiles. Of these, 421 belonged to SB0120, 103 to SB0134, and 61 to SB0841. Despite the high degree of resolution (H index >0.98) (Boniotti et al., 2009), twelve genotypes belonging to SB0120 were found in more than 56 herd outbreaks, mainly located in Sicily, Campania, and Apulia since 2008 (Supplementary Tables S1, S3).

Among them, the most prevalent was the genetic profile VNTR-MIRU 4,5,5,3,3,10,4,4,4,3,6,5, as described in M&M section (Italian genotype 1), which was identified between 2008–2020 in 190 cattle/buffalo herds (5,6% of outbreaks). This genotype was widespread in different regions (Veneto, Campania, Marche, Lazio, Apulia, Calabria, Sicily), and was the cause of several herd outbreaks localized in a specific geographical area of Sicily (Caronia, province of Messina). The second most frequent profile belonging to SB0120 was VNTR-MIRU 3,3,5,3,3,10,4,4,4,3,6,5 (Matelica Clade), which differs from the Italian genotype 1 in the ETRA and ETRB loci (3 repeats instead of 4 and 5, respectively). The Matelica Clade has been isolated in 111 cases of bTB in cattle herds and wild boars (77), mainly in the hunting district of Monte San Vicino, within the municipality of Matelica (Macerata, Marche region, Central Italy). Since the first isolate of *M. bovis* was detected in a hunted wild boar in 2002, the same genetic strain has been circulating in this area. Several bTB outbreaks were detected in cattle herds in the following years, as well as in wild boars, raising suspicion of a wildlife-livestock interface. Out of the total cases, we were able to sequence only 17 samples with high-quality DNA; retrieving DNA from samples collected during the 2010–2017 period, especially before 2010, proved to be difficult.

### 3.2 Italian SB0120 *Mycobacterium bovis* population in the international context

Since little is known about the global lineages of Italian *M. bovis* genotypes, a comparative SNP analysis was performed, including 81 sequences from Italy, 76 SB0120 isolates of the Italian Genotype 1 and Matelica Clade, and 5 outgroups (see Materials and Methods section). Additionally, a selection of 100 sequences covering the European genetic diversity of *M. bovis* (see Supplementary Table S2) (Hauer et al., 2019, Kohl et al., 2020, Loiseau et al., 2020, Zwyer et al., 2021) was added to the SNP analysis. In particular, we included the well-known *M. bovis* clonal complexes EU1 (La 1.8.1) and EU2 (La 1.7.1), the new *M. bovis* sub-lineages Unknown 7 (La 1.8.2) and Unknown 3 (La 1.4) described by Zwyer et al. (2021) and Loiseau et al. (2020), which showed a broad distribution in Europe and Asia, South America and Asia, respectively. Furthermore, based on the studies of Zwyer et al. (2021), and Loiseau et al. (2020) we selected several genomes of different geographical origins characterized by spoligotype SB0120 and belonging to the La 1.2 clade, which corresponds to the Unknown 2 described by Loiseau et al. (2020) and to the EU3 described by Branger et al. (2020). *M. caprae* isolates (La2) were included in the analysis as additional outgroups. Among the international genomes selected for this study, we included a unique Italian isolate from human (SRR7131117, Loiseau et al., 2020) that did not cluster with newly sequenced Italian isolates, but with French and Swiss isolates (minimum pairwise SNPs with SRR7131117 = 104 SNPs) (Supplementary Table S5).

All Italian sequenced isolates belonged to La1.2 lineage and exhibited SNPs specific to La1.2, as defined as by Zwyer et al. (2021) except for 232080–2010 sample as resulted in KvarQ analysis (Supplementary Table S4). In this study, the maximum pairwise genetic distance among Italian isolates of the La1.2 lineage was 319 pairwise SNPs (Supplementary Table S5), while the highest pairwise SNPs difference with international isolates of different La lineages was 1,080 pairwise SNPs.

**Figure 1:**
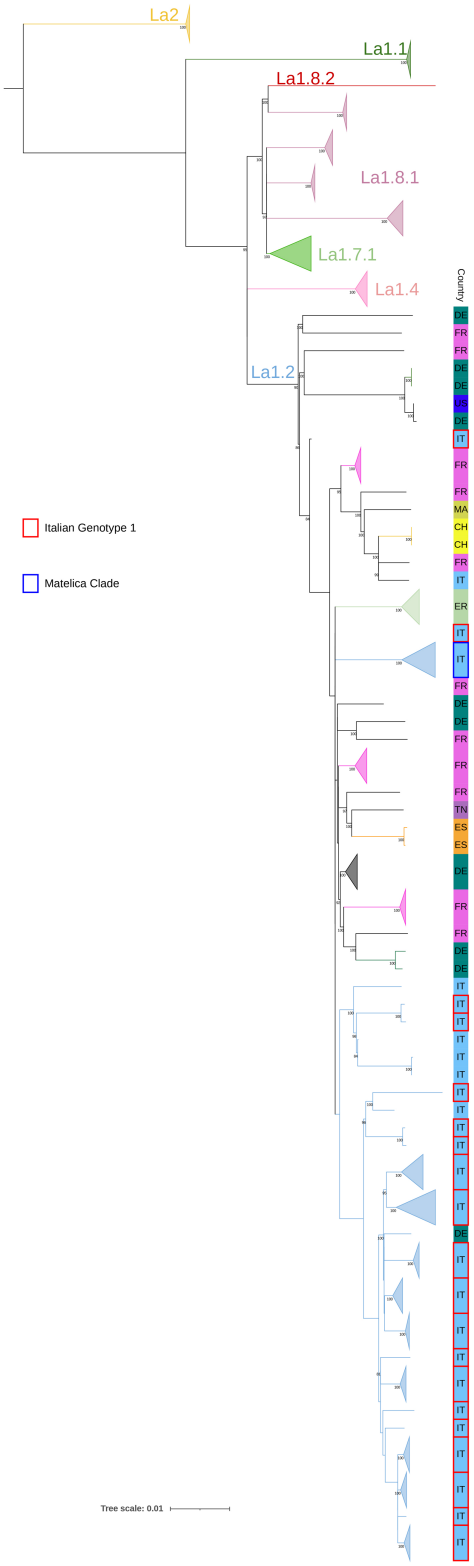
SNP based phylogenetic analysis of the *M. bovis* population. Lineages, previously described by Zwyer et al. (2021), are reported. Isolates belonging to the Matelica Clade and the Italian Genotype 1 are indicated in blue and red, respectively (see legend). Isolates of La1.2 lineage of same Country and type of Clade with 100% bootstrap values (>3 isolates) are collapsed. Other European strains are made explicit in the figure and their international acronym is reported. Bootstrap values higher than 80% were shown near their relative nodes.

In the context of La1.2 lineage, Italian isolates of La1.2 clustered separately from other European strains included in the study, except for one isolate from Germany (ERR2815526, Kohl et al., 2020) that shared a minimum of 82 pairwise SNPs with the nearest Italian genomes. In particular, apart from the isolates of the Matelica Clade, all the Italian isolates of Genotype 1 clustered in a single polyphyletic clade that also comprises outgroup isolates with different spoligotype and VNTR-MIRU profiles (Figure 1). Two isolates (232080–1-2010 and 217495–8-2008) lay outside this polyphyletic clade: 232080–1-2010 was not classified as a La 1.2 strains, while 217495/8 diverged from the other isolates in this study (pairwise SNPs range = 1–140 with an average number of SNPs of 72), despite their belonging to the same Spoligotype, VNTR-MIRU profile and La1.2 lineage (Supplementary Table S5); this could be possibly explained with convergent evolution events of VNTR markers, already observed in *Mycobacterium tuberculosis* (Comas et al., 2009). This last strain will be excluded from SNP metrics associated with the Italian La1.2 isolates, ensuring that genetic variability is assessed solely based on point mutations.

### 3.3 WGS analysis of *Mycobacterium bovis* Italian genotype 1 and Matelica Clade

**Figure 2:**
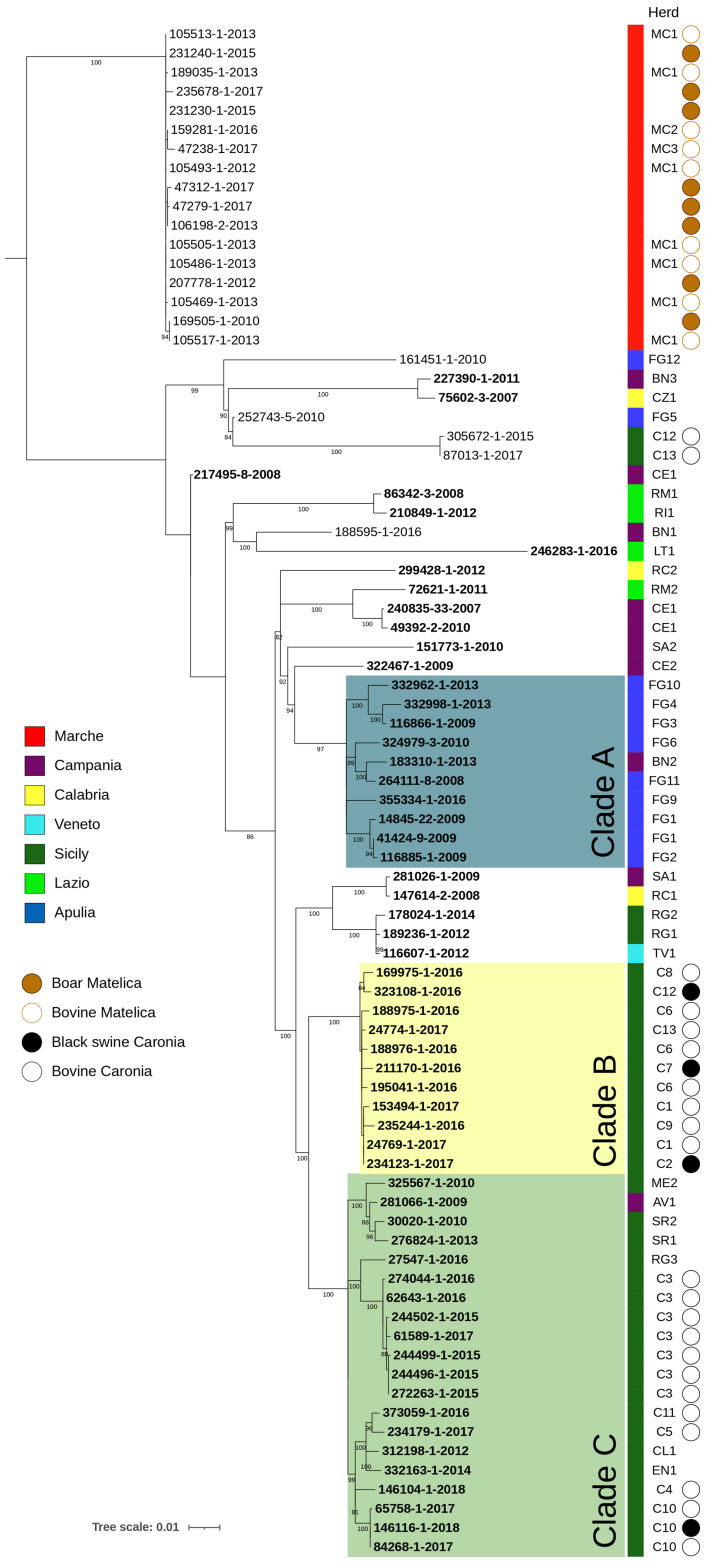
SNP based phylogenetic analysis of the Italian *M. bovis* strains considered in this study. Colored squares indicate the belonging to different Italian regions, as reported in the legends. The herd column highlights the herd to which each isolate comes from. Isolates from black swine collected from Caronia are marked with a bold black circle, while bovine isolates from the same area are reported with an empty circle. The Matelica column contains a bold brown circle figuring out boar isolates, while an empty circle indicates bovine isolates. Bootstrap values higher than 80% were shown near their relative nodes.

A second phylogenetic analysis was performed on *M. bovis* Italian isolates sequenced in this study and outgroups described in the Materials and Methods section (Figure 2). This analysis aims to evaluate the genetic diversity within and between Italian regions (corresponding to the Nomenclature of Territorial Unit for Statistics 2 level), as well as within individual herds and between epidemiologically correlated herd outbreaks.

As expected, isolates belonging to Italian Genotype 1 and Matelica Clade, which differed in ETRA and B loci, clustered separately with an average of 254 pairwise SNPs (min 239, max 334, median 259) (Supplementary Table S6). Within the Italian genotype 1, where the pairwise SNP differences ranged from 1 to 242 SNPs, sub-clusters were distinguishable, and they were generally associated with different Italian regions (shown with different colors in Figure 2). Clusterization by regions was more evident for Apulia (Blue) with almost all the isolates grouped in clade A (pairwise SNPs range = 2–50 SNPs) and for Sicily (dark green) where the majority of isolates grouped in clade B and C (max pairwise SNPs = 77 SNPs), which derived from the same evolutionary branch. The pairwise SNP differences between clade A (Apulia) and clade B + C (Sicily) ranged from 102 to 129 SNPs.

To evaluate the genetic diversity within epidemiologically correlated herd outbreaks, we considered 3 cases where the observed epidemiological links (i.e., animal movements between herds and geographical proximity), were supported by the genetic relationships among isolates: (1) Isolates of RM1 and RI1 herds from Lazio region (light green) showed few pairwise SNPs (10 SNPs), consistent with the transmission of bTB by the trading of an infected animal from RM1 to RI1 and with the evolution of the outbreak strain in five years; (2) FG2 isolate corresponded to an animal originating from the FG1 herd (SNPs = 7); and (3) TV1 isolate corresponded to an animal originating from the RG1 herd (SNPs = 3).

Within-herds genetic diversity was evaluated in 8 herds (C12, MC1, CE1, FG1, C6, C1, C3, C10) with 2–7 strains from each herd. Variation detected in isolates of the same herd (CE1, FG1) was, respectively, of 4 and 5 SNPs. Two isolates of C10 herd showed a strict genomic correlation (pairwise SNPs range = 0–1 SNPs) with a black swine isolate of the same herd, suggesting contamination among different animals of the same herd. Also, in C3 herd the spread of a single clone over years (2015–2017) was observed (pairwise SNPs range = 0–5 SNPs).

**Figure 3:**
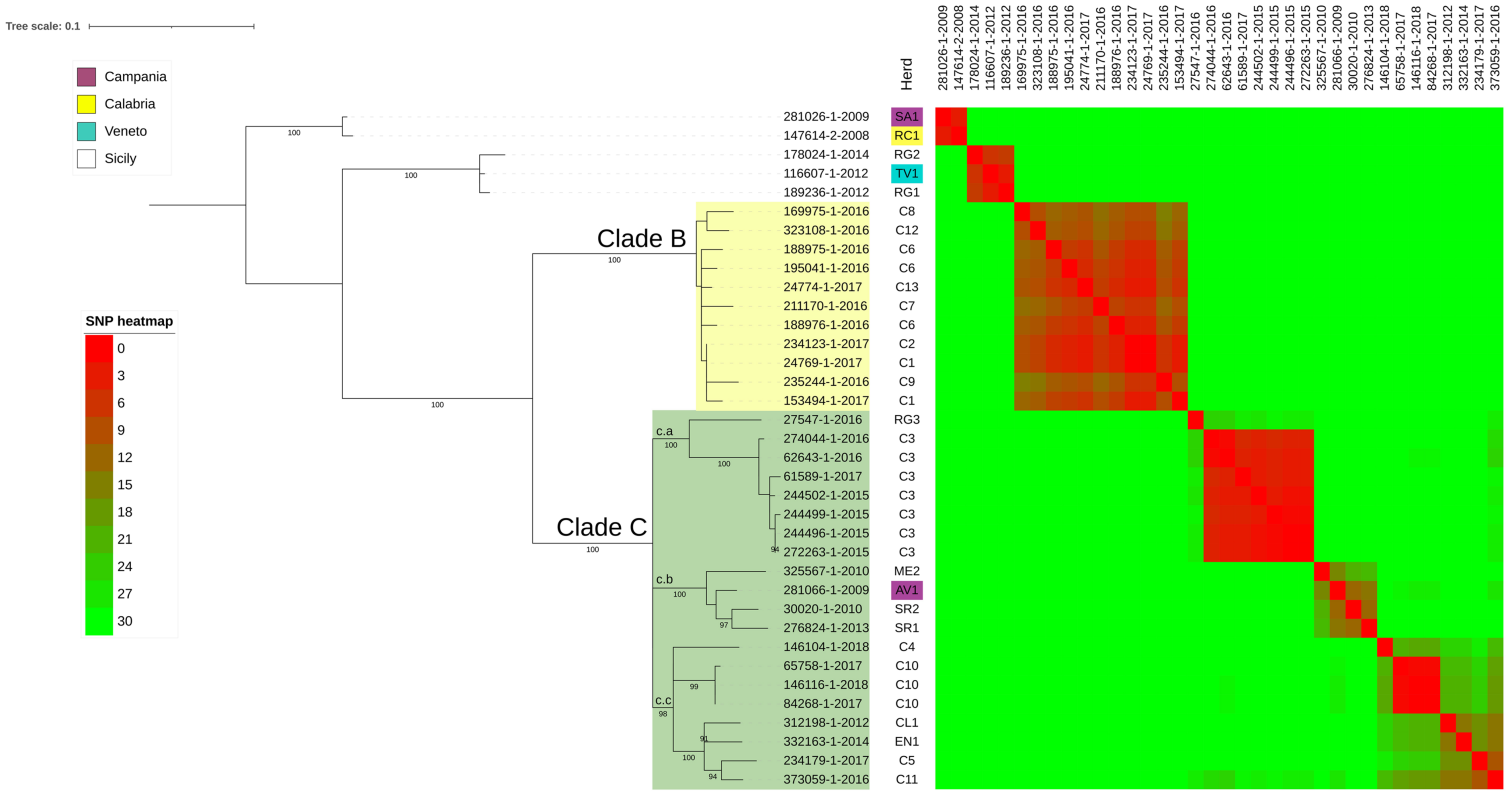
SNP based phylogenetic analysis of the node including all the Sicilian isolates. Herds of each isolate are shown in the first column and herds of region different from Sicily are colored following the legend. The heatmap represents the number of SNPs between each couple of isolates; heatmap colors are described in the legend. Bootstrap values higher than 89% were shown near their relative nodes.

Additionally, we evaluated the genetic diversity observed between bTB isolates originating from herds linked by geographical proximity. Specifically, we examined two scenarios:

1. Clade B analysis: this clade includes isolates from epidemiologically linked herds (C1, C2, C6, C7, C8, C9, C12, C13) located in Caronia municipality. In this clade, the pairwise SNPs ranged from 8 to 15 SNPs (see SNP heatmap in Figure 3). Notably, black swine isolates clustered alongside bovine isolates with a pairwise SNPs range of 3 to 12 SNPs (Figure 3). These herds, located within the Nebrodi Park, were epidemiologically correlated due to the sharing of grazing areas (Supplementary Figure S2);
2. Matelica clade analysis: the Matelica Clade consisted of 17 isolates collected between 2010 and 2017 (see Supplementary Table S1). This genotype included 9 isolates from three bovine herds (MC1, MC2, MC3) and 8 wild boars. All isolates grouped within a single cluster (Figure 2) with low SNPs diversity (pairwise SNPs range = 0–9 SNPs), thus supporting a geographical epidemiological correlation. The maximum SNP distance among isolates of the MC1 herd was three pairwise SNPs, while the distance ranged from one to seven SNPs among the three herds (MC1, MC2, and MC3), showing high genomic similarity in this geographic area. Moreover, wild boar isolates clustered together with bovine isolates (pairwise SNPs range among wild boar isolates = 0–7 SNPs; pairwise SNPs range among wild boar and bovine isolates = 0–9 SNPs). In particular, some wild boar isolates (namely 169505, 207778-20, 23230 and 231240) had no (0) SNPs diversity concerning MC1 isolates (105517–20, 105486–20, 105493–20, 105505, 105513). Isolates of the herd MC3 were more distant from wild boar isolates (pairwise SNPs range = 5–9 SNPs) and MC1 and MC2 bovine isolates (pairwise SNPs range = 4–7 SNPs) than the other herds. In general, despite the collection of these strains over time (seven years), the majority of wild boar isolates showed only a few SNPs of difference (median 2.5 SNPs).

**Figure 4:**
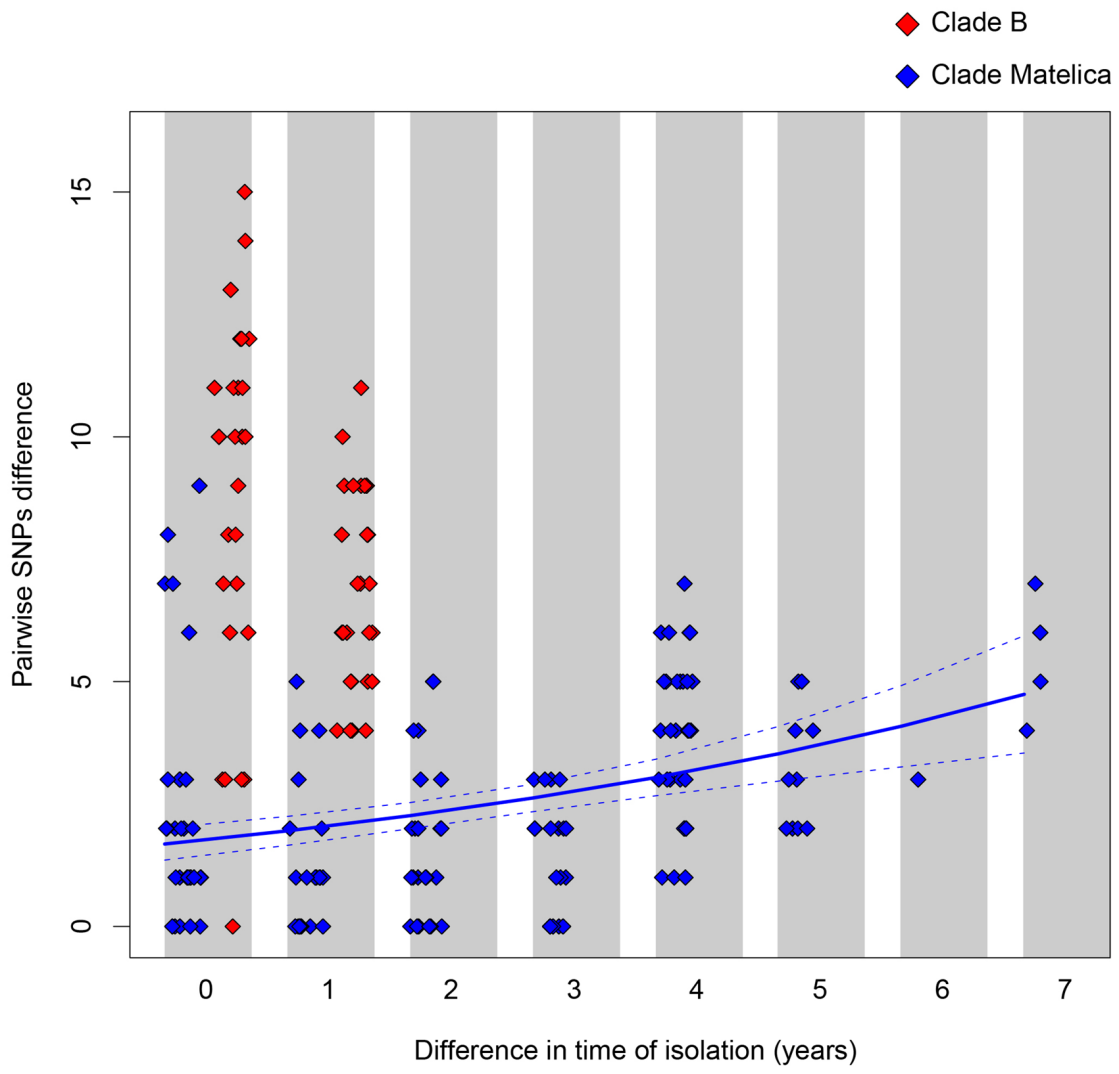
Pairwise SNPs differences between strains belonging to Clade B (red diamonds) and to the Matelica Clade (blue diamond) as a function of the time difference elapsed between the sampling dates. The solid blue line represents the pairwise SNPs difference as a function of the sampling date difference within the Matelica Clade estimated through a generalized linear model with Poisson distributed error. The dashed lines represent the confidence interval of the estimate.

Despite the longer time elapsed among the sampling date of the Matelica Clade strains with respect to the Clade B ones (seven vs. two years), the Matelica Clade showed a significantly smaller pairwise genetic variability than Clade B (generalized linear model, GLM, with Poisson distributed error, *p* < 0.0001). In addition, the strains in the Matelica Clade showed a significant increase in the pairwise genetic variability when the difference elapsed between the sampling dates of strains increased (GLM with Poisson distributed error, *p* < 0.0001), see Figure 4.

## 4 Discussion

In Italy, despite recent progress in the eradication campaign, bovine tuberculosis (bTB) continues to have a significant economic impact due to high control and eradication costs, compensation for slaughtered TB-positive animals, and trade restrictions on live animals and animal products. The new Animal Health Law (Regulation (EU) 2016/429, 2016) has introduced changes in disease management, including updated diagnostic protocols, risk assessment, and epidemiological investigations (European Food Safety Authority and European Centre for Disease Prevention and Control, 2022).

Molecular genotyping is essential for tracking infectious agents in epidemiological investigations. In bovine tuberculosis (bTB), techniques like whole-genome sequencing (WGS) offer insights into the pathogen’s evolution, transmission, and distribution. WGS surpasses traditional methods (Spoligotyping and VNTR-MIRU) by enabling rapid sequencing of entire bacterial genomes, thus improving epidemiological accuracy, especially in cases with frequent Spoligo-VNTR-MIRU profiles. Few Italian *M. bovis* genomes have been published, and there’s limited knowledge on the association of specific clonal groups with VNTR-MIRU genotypes in Italy (Hauer et al., 2019; Kohl et al., 2020; Loiseau et al., 2020; Zimpel et al., 2020). This study is the first large-scale genomic analysis of *M. bovis* in Italy, aiming to use SNP analysis from WGS to determine the phylogenetic relationships of the two most common SB0120-VNTR-MIRU profiles in Italy (Genotype 1 and Matelica Clade) and compare them with European sub-lineages. The study analyzed 81 *M. bovis* isolates, primarily from regions where bTB persists: Sicily, Campania, and Apulia.

Despite the presence of SB0120 in other lineages (Branger et al., 2020), all sequenced Italian isolates (Matelica Clade and Italian genotype 1) belong to La1.2 lineage (maximum pairwise genetic distance of 319 pairwise SNPs among Italian isolates of La1.2 lineage; Figure 1). These isolates clustered separately from European isolates, suggesting an Italian genomic segregation. Our SNP analysis confirms the high genetic variability within SB0120. For instance, the French reference strain Mb3601 (ERR3825346 in Figure 1; Branger et al., 2020) differs from 116 to 291 SNPs from isolates of the Italian Genotype 1. This variability aligns with findings observed by Hauer et al. (2019).

Another purpose of our study was to evaluate the genetic diversity within and between Italian regions, within epidemiological correlated outbreaks, and within herds. To assess genetic diversity within and between Italian regions, we considered 36 isolates from Campania, Sicily, Apulia, Calabria, Veneto, Lazio collected between 2008 and 2018. As expected, the phylogenetic analysis showed that isolates belonging to Italian Genotype 1 and Matelica Clade, which differ in ETR-A and -B loci, clustered separately (Figure 2), with an average genetic distance of 254 pairwise SNPs (min 239, max 334, median 259). Within the Italian genotype 1, which is the prevalent genotype in Italy, there is a genetic diversity ranging from 1 to 242 SNPs, indicating prolonged circulation across multiple regions. Sub-clusters were generally region-specific (i.e., Apulia in blue and Sicily in dark green in Figure 2), with high genetic variability within regions (pairwise SNPs range = 2–70 SNPs).

A recent study in Spain (Pozo et al., 2022), on *M. bovis* isolates from different host species, including cattle, wild boar, and red deer, revealed the genetic distance was primarily geographic rather than host-specific. Isolates from both cattle and wildlife from a given region were more closely related than isolates from the same species but geographically distant. Moreover, the genetic diversity among the sequenced isolates, all belonging to the SB0339 spoligotype, ranged from 49 to 88 SNPs. These values are lower than the ones observed for the Italian genotype 1 (pairwise SNPs range = 1–242), all belonging to the same VNTR-MIRU profile, suggesting longer circulation of Italian genotype 1 in Italy than Spanish isolates.

To asses genetic diversity within outbreaks in geographically close areas, two main groups of isolates were considered: (i) 24 isolates from cattle and black pigs in a delimited *M. bovis* hot-spot area in Sicily (Caronia, Messina province) with known epidemiological information over three years (2015–2018) and (ii) 17 isolates, belonging to Matelica Clade, from cattle and wild boar collected from 2010 to 2017.

The isolates located in the Caronia municipality clustered into clades Clades B and C (Figure 3), with Clade C showing three sub-clusters (c.a, c.b and c.c) originating from a common ancestor, and including isolates from other areas of Sicily, thus suggesting a large-scale circulation of these lineages in Sicily. While the co-circulation in the Caronia municipality of different Spoligotype-VNTR-MIRU profiles has been already documented, suggesting multiple introductions of bTB in the area (Marianelli et al., 2019, 2023), here we showed that strains belonging to the same Spoligotype-VNTR-MIRU profile originated from distinct introductions.

On the other hand, Clade B was associated with peculiar geo-epidemiological and managerial characteristics of the infected herds. Eight cattle herds fall within Clade B (C1, C2, C6, C7, C8, C9, C12, C13), all with established epidemiological connections due to direct and indirect mutual contacts. These farms are located within the Nebrodi Park, a large nature reserve of about 86,000 hectares, where bTB is historically endemic. The park’s diverse wildlife, including foxes, wild boars, martens, and fallow deer, shares feeding and watering points with livestock, facilitating disease transmission. Notably, the feral and free-roaming pigs dominate in terms of population density among these species.

Herds in Clade B are located within a 20 km^2^ area in 2 geographic areas: the first (C8, C12, C1, C6, C13) with an average inter-herd distance of about 1.5 km, while the second (C2, C6, C7, C9), 5 km away, with an average inter-herd distance of 2.1 km. Although these farms have distinct registration codes, they constitute a single epidemiological unit due to consistent direct and indirect interactions over time and space. Operating as mixed farms, hosting cattle, pigs, sheep, goats, donkeys, and horses, these farms practiced extensive grazing, with minimal shelter and fencing, leading to constant animal-wildlife contact, especially at shared water and foraging sites (Fine et al., 2011). Marianelli et al. (2023) highlighted that TB spread is only limited by natural geographic barriers without appropriate biosecurity measures. Moreover, studies in the Nebrodi Park suggested that the Black Nebrodi pig, typically raised in a free-roaming system, could act as a bTB reservoir (Di Marco et al., 2012).

The Matelica Clade isolates, including 9 isolates from three bovine herds (MC1, MC2, MC3) and 8 wild boars from 2010 to 2017, formed a single clonal cluster (pairwise SNPs range = 0–9 SNPs) (Figure 2), supporting the geographical epidemiological correlation. This indicates an eight-year contamination period in this area, with MC1 and 4 wild boars isolates showing no genetic differences. Epidemiological investigations revealed numerous epidemiological connections between MC1 and MC2, including shared pastures for several years, while MC3 introduced bTB via a bovine bought from a herd located in the same province, but geographically more distant from MC1 and MC2. However, all herds had possible contact with infected wild boars either on the pasture (MC1 and MC2) or at the cattle shed.

In this area, the same genetic strain has been circulating since 2002, when the first isolation of *M. bovis* from a hunted wild boar occurred (unpublished data). Since then, many bTB outbreaks have been detected in cattle herds accompanied by a notable rise in *M. bovis* isolations within wild boar populations, suggesting a wildlife-livestock interface (Figure 2). In this region, native beef cattle practice seasonal migration to shared pastures and are maintained in semi-free stabling, fostering interactions with wild boar. Moreover, the lack of biosecurity measures to protect herds from contact with wild animals has escalated the spread of bTB outbreaks among cattle herds and spread the disease within the wild boar population across the Marche region.

Notably, both Matelica and Caronia cases showed clustering of isolates from different species with bovine isolates (Figures 2, 3), suggesting repeated transmission between cattle and wild boar or black pig. As reported by other studies (Johnston et al., 2011; Martínez-López et al., 2014) the transmission, diffusion, and persistence of bovine tuberculosis within and between species are closely related to risk factors such as shared pasture, presence of feeding sites, and the absence of physical barriers. The transmission of bTB in wildlife raises public health concerns, including risks for hunters handling infected game meat and consumers of potentially contaminated wild boar products. Our NGS findings highlight the need for veterinary and public health authorities to develop specific and dedicated bTB control plans for geographical areas where bTB is present in wild animals or in multi-host context.

By comparing the genetic findings in Clade B and Matelica Clade, we observed a larger variability in pairwise SNP differences between isolates belonging to Clade B with respect to the variability observed between isolates belonging to the Matelica Clade (see Figure 4). While the genetic variability observed within the Matelica Clade is similar to those observed in mycobacteria outbreaks investigated through WGS (see Biek et al., 2012; Trewby et al., 2016; Bolzoni et al., 2021), the genetic variability observed within Clade B in Caronia municipality is higher than expected. The higher genetic divergence between isolates in Clade B can be explained by hypothesizing an older common ancestor of Clade B, suggesting a really old introduction of this bTB clade in the Caronia area.

Finally, the genetic diversity within 7 herds (MC1, CE1, FG1, C6, C1, C3, C10) showed persistence of clones within herds, with SNP pairwise differences ranging from 0 to 7 SNPs. There was a strict genomic correlation among isolates of different species within these herds, such as black swine and bovine isolates from C10 (0–1 SNPs), and among clones over several years, such as C3 with isolates from 2015 to 2017 (0–5 SNPs). Our data differ from those published by Pozo et al. (2022) in Spain, where genetic distance within herds ranged between 0 and 44 SNPs. However, in our study, we considered only isolates with the same Spoligo-VNTR-MIRU profile, resulting in a reduced genetic distance.

Next, Generation Sequencing is rapidly becoming the gold standard for molecular genotyping of human and bovine tuberculosis. This powerful technology enables the sequencing of entire bacterial genomes, providing unprecedented resolution in the identification and tracking of infectious agents to their source and transmission routes. However, to fully leverage the potential of NGS in tuberculosis genotyping for a One Health approach, it is essential to ensure comparability of data across different studies and regions. This requires the implementation of standardized protocols and the establishment of a global database, allowing for cross-species comparisons and contributing to a more integrated and comprehensive approach to disease surveillance, prevention, and control. The use of NGS and the establishment of standardized protocols and databases will be key in the fight against tuberculosis, enabling a more effective One-Health approach to tackle this disease that affects both animals and humans.

## Data availability statement

Raw reads of isolates described in this article were submitted to EBI-ENA (https://www.ebi.ac.uk/ena/browser/home) under study accession n. PRJEB46575 (https://www.ebi.ac.uk/ena/browser/text-search?query=PRJEB46575).

## Ethics statement

Ethical approval was not required for the studies on animals in accordance with the local legislation and institutional requirements because only commercially available established cell lines were used.

## Author contributions

ES: Conceptualization, Data curation, Methodology, Writing – original draft. KI: Investigation, Methodology, Writing – original draft. MB: Conceptualization, Data curation, Methodology, Writing – original draft, Writing – review & editing. IM: Data curation, Investigation, Methodology, Writing – original draft. LB: Data curation, Writing – review & editing. DI: Investigation, Methodology, Writing – original draft. FC: Investigation, Methodology, Data curation, Writing – review & editing. DL: Investigation, Methodology, Writing – original draft. MD’I: Investigation, Methodology, Writing – original draft. MZ: Investigation, Writing – review & editing, Data curation, Methodology. VP: Investigation, Writing – review & editing, Conceptualization, Supervision. PM: Investigation, Writing – review & editing, Methodology. SG: Writing – review & editing, Data curation, Investigation. MP: Writing – review & editing, Conceptualization, Funding acquisition, Project administration, Supervision, Writing – original draft.

## Footnotes

^1^ http://www.pasteur-guadeloupe.fr:8081/SITVIT_Bovis

^2^ https://itol.embl.de/
