# Supplementary figures and images for: Inside Mycobacterium bovis SB0120 spoligotype circulating in Italy: analysis of the most frequent genotypes by whole genome sequencing
Source: Front Microbiol. 2024 Jul 26;15:1416605. doi: 10.3389/fmicb.2024.1416605 (PMC11310128; doi:10.3389/fmicb.2024.1416605)

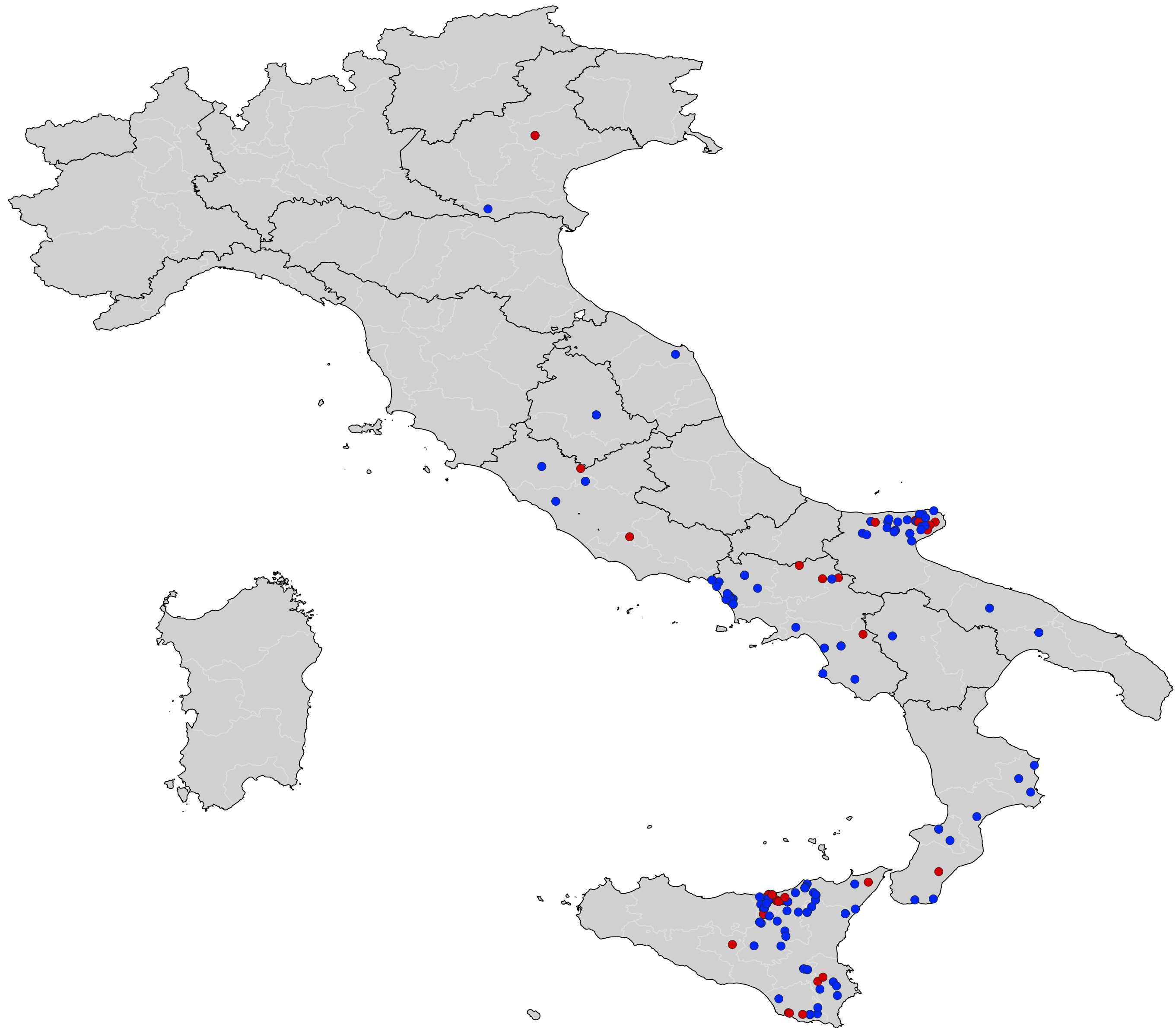

Supplement: Supplementary file 1 [file Data_Sheet_1.PDF]

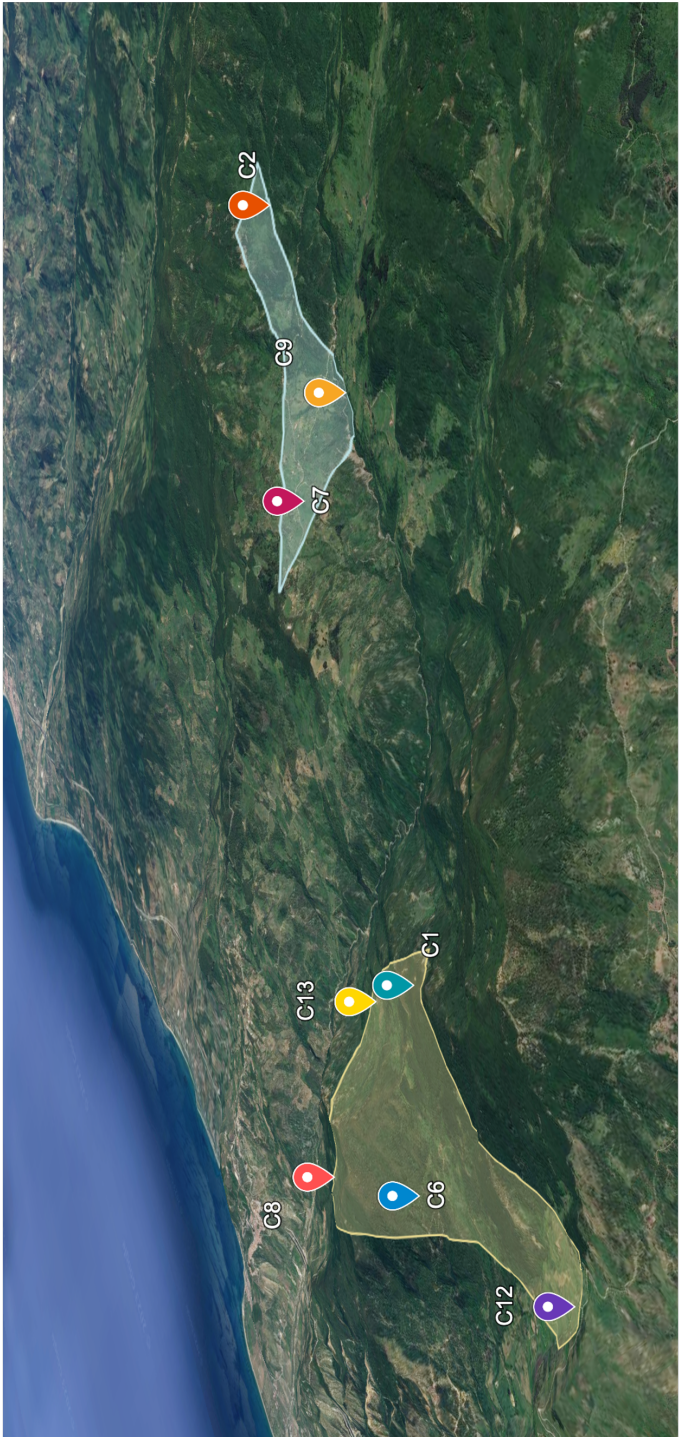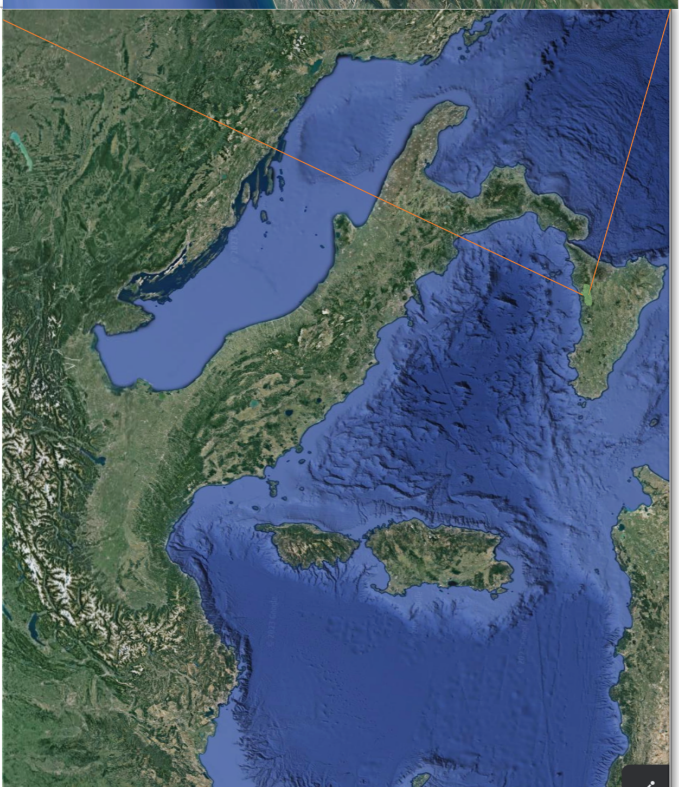

Supplement: Supplementary file 2 [file Data_Sheet_2.PDF]
