# Supplementary material for: Inside Mycobacterium bovis SB0120 spoligotype circulating in Italy: analysis of the most frequent genotypes by whole genome sequencing
Source: Front Microbiol. 2024 Jul 26;15:1416605. doi: 10.3389/fmicb.2024.1416605 (PMC11310128; doi:10.3389/fmicb.2024.1416605)
Supplement: Supplementary file 3 [file Presentation_1.pdf]

Supplementary material of the article: “Inside Mycobacterium bovis SB0120 spoligotype circulating in Italy: analysis of the most frequent genotypes by Whole Genome Sequencing”

Supplementary Figure Legends

Supplementary Figure 1: **Distribution of Italian Genotype 1**. Geographical distribution of strains belonging to genotype SB0120 and MLVA 4,5,5,3,3,10,4,4,4,3,6,5, collected between 2008 and 2018. Red dots represent isolates sequenced in this study, while blue dots indicate isolates that were not sequenced.

Supplementary Figure 2: **Distribution of Clade B isolates.** Satellite images of the Caronia area (right picture). The localization of herds belonging to Clade B is indicated by their codes.
